# Assessing the Antibiotic Resistance in Food Lactic Acid Bacteria: Risks in the Era of Widespread Probiotic Use

**DOI:** 10.1002/fsn3.70740

**Published:** 2025-07-31

**Authors:** Salma Sherif Refaat, Zeynep Akinan Erdem, Muhammed Zahid Kasapoğlu, Fatih Ortakcı, Enes Dertli

**Affiliations:** ^1^ Department of Food Engineering Faculty of Chemical and Metallurgical Engineering, Istanbul Technical University Istanbul Turkey; ^2^ Department of Nanotechnology Institute of Nanotechnology and Biotechnology, Istanbul University‐Cerrahpaşa Istanbul Turkey

**Keywords:** antibiotic resistance, biotherapeutics, food, lactic acid bacteria, mitigation strategies, probiotics

## Abstract

Antibiotic resistance (AR) in lactic acid bacteria (LAB) has become an emerging concern in the probiotic and food industries. LAB, a key component of the human microbiota and widely used in probiotic products, can harbor antibiotic resistance genes (ARGs), which may transfer to pathogenic microorganisms. This review provides an updated overview of AR in LAB, outlining mechanisms of resistance, both intrinsic and acquired, and their implications for probiotics. Recent studies have reported the presence of resistant LAB strains in food products, highlighting the need for stringent monitoring and regulatory measures. Additionally, the review addresses the declining production of new antibiotics, exacerbating the AR crisis. Advances in bioinformatics offer powerful tools for predicting and identifying ARGs in LAB, providing powerful tools to combat this issue. We also discuss strategies to mitigate AR in LAB, such as the use of nanotechnology, combination therapy, bacteriocins, and the potential role of CRISPR and other genome editing tools.

## Graphical abstract

Targeting the potential sources and risk factors for antibiotic resistance transmittance and development of antibiotic resistant‐free probiotics.

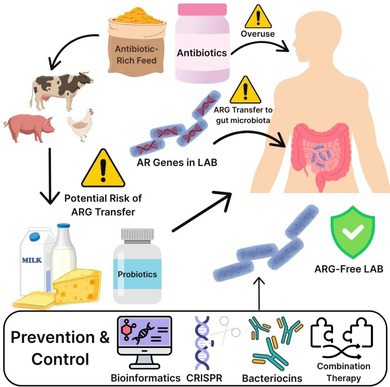

## 1 Introduction

Lactic acid bacteria (LAB) are a group of gram‐positive bacteria that have a widespread presence in the food industry, particularly in the fields of food fermentation, bioprocessing, preservation, and as feed additives (Punia Bangar et al. 2022). Due to their ability to successfully transform sugars into lactic acid, LAB have been instrumental since ancient times and continue to play a key role in the manufacturing of common fermented foods, such as various cheeses, yogurt, kefir, sourdough bread, and wine (Kulakauskas 2014; Ter et al. 2024). Through fermentation, these bacteria enhance food safety by inhibiting the growth of harmful pathogens, primarily by producing organic acids and bacteriocins (natural antimicrobial peptides) (Icer et al. 2023). Additionally, certain LAB, such as *Lactobacilli*, act as probiotics in the aforementioned fermented foods and animal feed, where they have been found to provide a wide range of beneficial effects on human or animal hosts (Zhang et al. 2020). The beneficial effects of LAB as probiotics in humans include enhancing gut health, modulating the immune system, improving conditions such as diabetes and cardiovascular diseases, reducing lactose intolerance, possessing antimicrobial and antioxidant properties, and supporting digestive health and overall well‐being (Barus et al. 2020; Hadjimbei et al. 2022; Hammam and Ahmed 2019; Huligere et al. 2024; Ibrahim et al. 2023).

The most important features of LAB starter cultures in foods are their swift lactic acid production, which initiates the fermentation process and improves the flavor profile of the treated foods (Blaya et al. 2018). The improvements in the sensorial quality and shelf life of food products can be achieved through the biosynthesis of compounds like B‐group vitamins (B1, B2, B6, B9, B12), gamma‐aminobutyric acid (GABA), and enzymes such as amylase and lactase (Icer et al. 2023). For example, GABA enhances the functional characteristics of food products such as dairy, cereal‐based items, and fermented beverages by improving their taste, texture, shelf life, and nutritional quality; additionally, it is capable of reducing blood pressure, relieving stress, improving sleep quality, and supporting neuroprotection (Icer et al. 2024). LAB can also survive in the acidic environment of the gastrointestinal tract and adhere to the intestinal lining, prolonging their beneficial effects in the gut (Dempsey and Corr 2022). These properties make LAB valuable functional starter cultures for controlled, industrial‐scale fermentation processes, with strains like *Lactobacillus delbrueckii* , *Lactiplantibacillus plantarum*, and *Weissella* species commonly used (Anumudu et al. 2024).

However, despite being deemed safe for human and animal consumption, as attested by their Qualified Presumption of Safety (QPS) and Generally Regarded as Safe (GRAS) status regulated by the European Food Safety Authority (EFSA) and the United States (US) Food and Drug Administration (FDA) (Ayed et al. 2024), LAB are increasingly recognized as potential reservoirs of antibiotic resistance (AR) genes, complicating the treatment against infections caused by pathogenic bacteria (Nunziata et al. 2022) (Table 1).

**TABLE 1:** Key definitions.

Term	Definition	References
Intrinsic resistance	Refers to the innate ability of a bacterial species to withstand the effects of certain antibiotics, due to inherent structural or functional characteristics, such as impermeable cell membranes, efflux pumps, or absence of the drug target	Poulton and Rock (2022)
Acquired resistance	Refers to the ability of bacteria to develop resistance to antibiotics that they were previously susceptible to, typically through two main mechanisms: horizontal gene transfer and spontaneous mutations in chromosomal genes	Blair et al. (2015); Kumawat et al. (2023)

Intrinsic antibiotic resistance in LAB provides a survival advantage to the gut microbiota when administered with certain antibiotics that may be used to combat ailments. The problem intensifies when LAB acquire antibiotic resistance through plasmid acquisition, horizontal gene transfer (HGT), or mutations (Wong et al. 2015). Among these, HGT plays a critical role by enabling the spread of resistance genes between bacterial species, particularly under stress conditions like antibiotic exposure (Liu et al. 2021). This is particularly concerning given the widespread presence of LAB in commercial food products. For instance, Heydari et al. (2021) found that Iranian commercial yogurt contains more than 100 million viable LAB per gram. Similarly, a granule type product contains six kinds of viable LAB, exceeding 10^8^ CFU/g, and a capsule‐type product contains 50 million viable LAB per capsule. These high concentrations of LAB in food products mean that any resistant strains can easily serve as reservoirs for AR genes, increasing the risk of those genes being transferred to the pathogenic bacteria in the human gut or during food processing and storage (Salvetti and O'Toole 2017). Notably, a 2023 systematic review by Shahali et al. (2023) of *Lactobacillus* in probiotic supplements and fermented foods identified instances of horizontal transfer of AR genes (such as tet and erm) from probiotic *Lactobacillus* strains to pathogens in at least nine studies, including both in vitro and in vivo settings.

Traditionally, fermented foods are produced through back‐slopping, which involves inoculating new batches with samples from previous batches. This method often leads to inconsistent product quality and can exacerbate the issue of AR spread since it is still in use for some homemade fermented products (Vinayamohan et al. 2023; Whittington et al. 2019). This practice may select LAB strains that carry antibiotic resistance genes, further increasing their prevalence in the food supply. Although defined‐strain and mixed‐strain starter cultures allow for greater control in production, they do not completely eliminate the risk of antibiotic resistance (Sharma et al. 2014). For example, well‐characterized strains from genera like *Lactobacillus*, *Streptococcus*, and *Bifidobacterium* have been shown to harbor resistance genes (Nunziata et al. 2022). In particular, some strains of *Enterococcus*, despite their role in food fermentations, are known to carry resistance traits, making their use in food products a potential contributor to the broader issue of antibiotic resistance (Belloso Daza et al. 2022). When LAB harbor these resistance traits, there is a risk they could transfer them to pathogenic bacteria (Twomey et al. 2002). Such horizontal gene transfer not only threatens the effectiveness of natural antimicrobials like bacteriocins, but also undermines food safety efforts. Given the widespread use of LAB in products such as processed cheese, dairy items, and canned foods, the potential for AR genes to disseminate through the food chain represents a significant public health concern.

As noted, when ingested in sufficient numbers, live LAB probiotic cultures, such as *Lactococcus*, *Lactobacillus*, *Bifidobacterium*, *Enterococcus*, *Bacillus*, and *Streptococcus*, have been shown to offer beneficial impacts to humans and animals (Huys et al. 2013). As a result, probiotic products are widely used in food, dietary supplements, infant formula, cosmetics, pharmaceuticals, and medical foods (Quin et al. 2018). Certain LAB are also utilized as direct‐fed probiotics to enhance animal production by promoting a balanced and beneficial intestinal microbiota, thereby improving the health and performance of animals (Das and Goyal 2012). In addition, the probiotic market reflects the growing recognition of their health benefits, which is projected to expand from 60 billion USD in 2021 to 90 billion USD by 2026, driven by a compound annual growth rate of 8.3% (Liang et al. 2024). Probiotics form a major part of the digestive product category within the over‐the‐counter (OTC) sector, which is the fourth‐largest OTC category globally (Sehrawat et al. 2022). This strong market performance highlights the increasing consumer demand for probiotics in managing digestive and other health‐related conditions. In this context, LAB play a crucial economic role in both the food and feed industries, highlighting their significant impact on improving health and productivity in agricultural settings.

Table 2 provides an overview of various LAB species that have been isolated from popular probiotic food sources, along with the associated health benefits they offer. By listing the LAB species and detailing their probiotic effects, this table illustrates the benefits of incorporating these probiotics into the diet, as well as emphasizes the importance of LAB in both food production and human health.

**TABLE 2:** Examples of popular probiotic sources, LAB species present in them, and their health benefits.

Food source	LAB species	Health benefits	References
Probiotic yogurt	*Bifidobacterium lactis* , *Lactobacillus acidophilus* , *Lacticaseibacillus casei*, *Lactobacillus delbrueckii ssp*. *bulgaricus*, *Lacticaseibacillus casei paracasei*, *Streptococcus thermophilus*	Benefits gut health, modulates the immune system, and helps with conditions like diabetes, cardiovascular diseases, and osteoporosis	Hadjimbei et al. (2022); Ibrahim et al. (2023)
Cheese	*Lactobacillus delbrueckii ssp*. *lactis*, *Lacticaseibacillus rhamnosus*	Enhance gastrointestinal health, boost immunity, lower cholesterol, reduce lactose intolerance, and may improve oral health by decreasing dental caries and yeast infections	Hammam and Ahmed (2019); Ibrahim et al. (2023)
Probiotic dairy beverages	*Lactobacillus acidophilus* , *Lactiplantibacillus plantarum*, *Lacticaseibacillus rhamnosus GG*, *Lacticaseibacillus paracasei*, *Bifidobacterium animalis ssp*. *lactis*, *Bifidobacterium bifidum*	Promote gut health, correct digestive disorders, and have been used therapeutically for conditions like tuberculosis	Turkmen et al. (2019)
Kumis	*Lactobacillus delbrueckii ssp*. *bulgaricus*, *Lactiplantibacillus plantarum*, *Lactobacillus helveticus*	Regulates blood pressure, improves immune health, good effect on the kidneys, liver, endocrine glands, gut system, nervous and vascular systems, treatment of digestive diseases	Afzaal et al. (2021)
Kefir	*Lactobacillus delbueckii ssp*. *lactis*, *Lactobacillus delbrueckii ssp*. *bulgaricus*, *Streptococcus thermophilus*	Lowers cholesterol levels, reduces lactose intolerance, and possesses antimicrobial and anticarcinogenic properties	Egea et al. (2022); Ibrahim et al. (2023)
Water kefir	*Lentilactobacillus hilgardii*, *Schleiferilactobacillus harbinensis*, *Lactobacillus satsumensis* , *Lactobacillus zeae*	Antioxidant, anti‐inflammation, healing activities	Kumar et al. (2021)
Sauerkraut	*Leuconostoc mesenteroides* , *Lactiplantibacillus plantarum*, *Lacticaseibacillus paracasei*, *Weissella cibaria*	Antioxidant, antimicrobial	Yang et al. (2020)
Kimchi	*Limosilactobacillus fermentum*, *Limosilactobacillus reuteri*, *Lacticaseibacillus rhamnosus*, *Lacticaseibacillus paracasei*, *Ligilactobacillus salivarius*	Acid‐bile tolerance, intestinal adhesion, cholesterol‐lowering ability, anti‐inflammatory activity, antimicrobial activity	Seo et al. (2021)
Tempeh	*Ligilactobacillus agilis*, *Limosilactobacillus fermentum*, *Pediococcus pentosaceus* , *Weissella confusa* , *Lactobacillus delbrueckii*	Prevent diarrhea and anemia, support gut health, and antioxidants	Barus et al. (2020)
Idli	*Lactiplantibacillus pentosus*, *Lactiplantibacillus plantarum*, *Limosilactobacillus fermentum*, *Lactobacillus delbreuckii ssp*. *lactis*, *Lactobacillus mesenteroides*, *Enterococcus faecalis*	Antimicrobial, antioxidant, source of vitamins, mainly B‐complex vitamins, proteins	Sircar and Mandal (2023); Shaikh et al. (2021)
Miso	*Bacillus subtilis* , *Enterococcus durans* , *Enterococcus faecium* , *Lactiplantibacillus plantarum*, *Pediococcus pentosaceus*	Improves gut health, and may offer anti‐diabetic, anti‐inflammatory, anti‐cancer, and protective cardiovascular effects	Allwood et al. (2021)
Sourdough bread	*Lactobacillus ssp*., *Enterococcus ssp*., *Weisella ssp*.	Improved glycemic control, reduced insulin response, increased satiety, enhanced nutritional quality, and support for gastrointestinal disorders like IBS and celiac sprue.	Pérez‐Alvarado et al. (2022)
Pickled vegetables	*Lactiplantibacillus plantarum*, *Levilactobacillus brevis*, *Leuconostoc mesenteroides* , *Pediococcus pentosaceus* , *Pediococcus acidilactici* , *Enterococcus faecium* , *Enterococcus faecalis*	Enhances immune resistance, prevents urogenital infections, suppresses cancer, improves digestion, and reduces serum cholesterol levels	Behera et al. (2020)
Pickled beetroot	*Lactobacillus delbrueckii ssp*. *lactis*, *Weissella cibaria*	High cholesterol removal, antioxidant activity	Maślak et al. (2022)
Pickled cucumber	*Lactiplantibacillus plantarum*, *Pediococcus pentosaceus*	Antioxidant, cholesterol assimilation, antibiotic susceptibility	Ahmed et al. (2021)
Natto	*Bacillus* (*subtilis*) *natto*, *Lactiplantibacillus plantarum*, *Lacticaseibacillus paracasei*	Lowering blood viscosity and fat, inhibiting platelet coagulation, lowering cholesterol, improving blood circulation, anticancer, antimicrobial, anti‐inflammatory, antioxidant	Chen et al. (2021); Chen (2021)
Gochujang	*Lactobacillus ssp*.	Antioxidants, immunomodulators	Jo and Hong (2021); Kim and Lee (2024)
Fruit vinegars	*Lacticaseibacillus paracasei*, *Lactiplantibacillus plantarum*, *Levilevilactobacillus brevis*, *Leuconostoc ssp*., *Weisella confusa*	Antimicrobial, antioxidative, antiglycemic, antiobesity, anticarcinogenic, antihypertensive, cardiovascular and lipid‐lowering effects	Sengun et al. (2022)
Kvass	*Lactiplantibacillus plantarum*, *Lactobacillus helveticus*	Antimicrobial, antioxidant, anti‐inflammatory, anticancer	Ekin and Orhan (2019); Pisponen and Andreson (2024)

In this context, the purpose of this article is to explore the critical relationship between probiotics, their regulation, and the rising threat of AR. As LAB gain prominence in the food industry for their health benefits, it is essential to understand how regulatory frameworks govern their use and safety. This article aims to highlight the challenges posed by AR in LAB, examining how the misuse of antibiotics can lead to resistant strains that compromise public health. Additionally various strategies that may be employed to mitigate AR in LAB, such as the use of advanced bioinformatic tools, the application of CRISPR technology, and antimicrobial nanomaterials are discussed in this study. By providing insights into current regulations and the implications of AR, the article seeks to inform researchers, regulators, and consumers about the importance of ensuring the safety and efficacy of probiotics while addressing the urgent need to combat the risks associated with AR.

## 2 Navigating the Safety of Probiotics and the Growing Danger of Antibiotic Resistance

### 2.1 Probiotic Regulation

There is an ever‐increasing demand for probiotics and their use has become widespread among consumers across all age groups. However, few of these commercially marketed products have been rigorously evaluated in controlled clinical trials and the majority of products make unproven efficacy claims (Arora et al. 2013; Lei et al. 2025; Liang et al. 2024). The regulatory approval of probiotic products before they are placed on the market is a significant concern involving several requirements (Garg et al. 2024). In particular, the quality, safety, and efficacy of these products are of great importance to those who produce and use them (Kumar et al. 2012; Liang et al. 2024).

When evaluating LAB safety and antibiotic resistance risks, it is crucial to recognize the potential influence of industry funding and conflicts of interest in published studies. Multiple meta‐analyses indicate that industry‐funded probiotic trials often report or actively seek results favorable to the sponsor's product, a phenomenon known as sponsorship bias (Hu et al. 2022; Saa et al. 2019). Such bias may influence study design, strain selection, MIC interpretation, and the disclosure of mobile resistance genes (Merenstein et al. 2023; Roe et al. 2022). Ethically sound probiotic research requires transparent guidelines, including: all funding sources and affiliations should be fully disclosed; standardized, independent safety protocols should be used; and, ideally, strains should undergo independent verification before commercialization. Furthermore, journals, regulators, and funding bodies should mandate registering all probiotic safety assessments and publish complete results to avoid publication and outcome‐reporting bias, ensuring public trust and safeguarding consumer health with unwavering ethical standards.

There is currently no unified global standard for determining the safety of probiotics in food and supplements (Gundogdu et al. 2023). As manufacturers introduce new species, strains, and next‐generation probiotics, the lack of universal guidelines could lead to inconsistencies in safety assessments. Various collaborators, such as the United States Pharmacopeia (USP), are actively working to establish leading practices for evaluating the safety and quality of these products (Roe et al. 2022).

**FIGURE 1:**
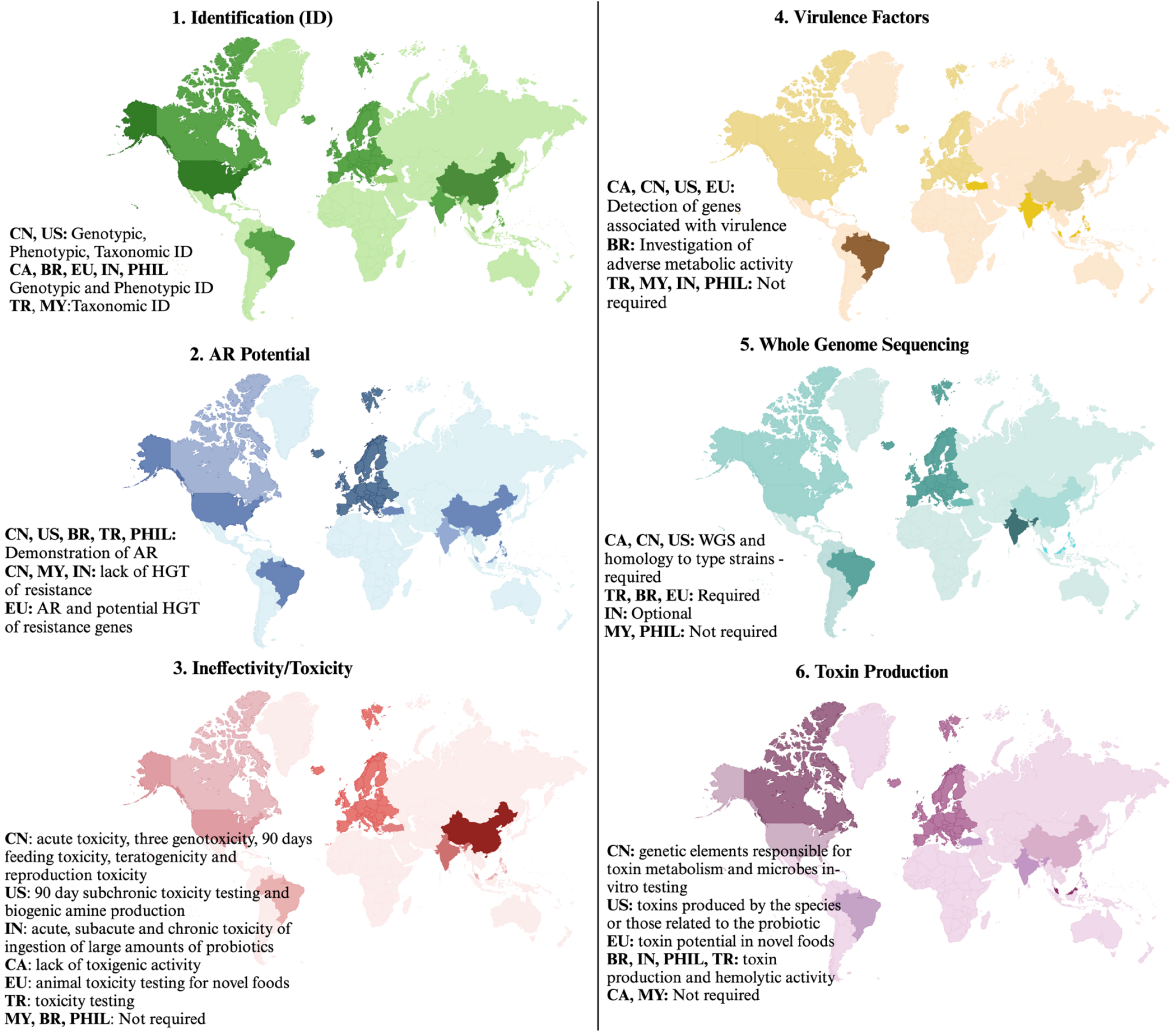
Regulatory requirements for probiotic bacteria in foods and supplements in different countries. Regulatory requirements for probiotic bacteria in foods and supplements in different countries (BR: Brazil, CA: Canada, CN: China, EU: European Union, IN: India, MY: Malaysia, PHIL: Philippines, TR: Turkey, US: United States) (Ministry of Agriculture and Forestry in Turkey 2024; Roe et al. 2022; Siong and Sum 2021).

The safety evaluation of probiotics consists of several crucial steps. To begin, strains are precisely identified using analytical methods, such as whole genome sequencing (WGS) and polymerase chain reaction (PCR) analysis, which confirm their uniqueness and highlight differences between strains (Duche et al. 2023). Additionally, it is essential for probiotic strains to be stored in recognized culture collections to maintain their genetic integrity and prevent changes over time (Roe et al. 2022). High‐quality annotated genome sequences are critical for assessing genetic components linked to antibiotic resistance, virulence, and toxin production. To support this process, the EFSA (2007) established a framework for evaluating the safety of microorganisms, focusing on taxonomic classification and specific testing at the strain level for antibiotic resistance, pathogenicity, and toxin production potential. Even for strains with a history of safe use, it remains important that evaluations also consider virulence and toxin gene assessments. Moreover, only probiotic strains that do not promote the transmission of antibiotic resistance should be used in food products. Evaluating antibiotic resistance involves phenotypic testing against clinically relevant antibiotics to determine the minimal inhibitory concentration (MIC) and identifying resistance genes through genomic analysis. According to EFSA (2021) and (EFSA 2023) guidelines, any resistance surpassing a specified threshold must be classified as either intrinsic or transmissible, along with an explanation of its genetic basis. Additionally, probiotics should be assessed for biogenic amines, which can be harmful in high amounts, and for factors like mucin degradation and the production of D‐lactic acid (Roe et al. 2022). Figure 1 outlines general regulatory requirements for probiotic bacteria used in foods or supplements across certain countries. When specified in guidelines or regulations, safety criteria may include aspects such as identification (ID), toxin production, WGS, the evaluation of virulence factors, infectivity, toxicity, and the appraisal of AR.

### 2.2 Growing Threat of Antibiotic Resistance in LAB

AR is defined by the capacity of bacteria and other microorganisms to encounter an antibiotic that was previously effective against them. The minimum inhibitory concentration (MIC) of a bacterial strain is a measure indicating the susceptibility of the bacteria to specific antibiotics, where a high MIC indicates greater resistance to the antibiotic (Brauner et al. 2016). The escalating prevalence of AR in infectious microbes can be traced back to the over prescription and misuse of antibiotics in various contexts, including medicine, animal feed, agriculture, and more. Specifically, human misuse of antibiotics may be regarded as the primary factor for the AR development (Velazquez‐Meza et al. 2022). Generally, AR arises from increased contact of the bacteria with the antibiotic, consequently developing the corresponding resistance. Furthermore, the outcomes of AR in humans include heightened rates of illness and death, prolonged hospital stays, contributing to extended treatment durations and escalated healthcare expenses (Muteeb et al. 2023). According to the World Health Organization's 2019 report, 700,000 people have died as a result of AR. Projections indicate that by 2050, this figure would rise to 20 million, with costs to cure anticipated to reach over $2.9 trillion (Watkins and Bonomo 2016).

Multidrug‐resistant bacteria (MDR), or “superbugs,” emerge due to genetic mutations and the overuse or misuse of antibiotics, which accelerate bacterial resistance (Van Goethem et al. 2024). This enables bacteria to survive multiple antibiotic treatments, evolving from single‐drug‐resistant to multi‐drug‐resistant, and in some cases, even pan‐drug‐resistant. Clinical misuse of antibiotics accelerates this process, leading to dangerous superbugs like vancomycin‐resistant enterococci, *Acinetobacter baumannii* , methicillin‐resistant *Staphylococcus aureus* , and *Pseudomonas aeruginosa* , which are significant causes of hospital‐acquired infections and are increasingly difficult to treat (Banin et al. 2017). Furthermore, the pharmaceutical industry has scaled back antibiotic research due to the rapid evolution of resistance and limited financial returns, causing innovation of new treatments to become increasingly challenging (Wasan et al. 2023).

## 3 Mechanisms of Antibiotic Resistance in Lactic Acid Bacteria

### 3.1 Mode‐Of‐Action (MOA) of Antibiotics and Their Relevance to AR in LAB

Antibiotics are among the most impactful medical innovations of the twentieth century, saving millions of lives since their discovery (Ribeiro da Cunha et al. 2019). Prior to the early 1900s, infectious diseases were the predominant cause of death. As an example, infectious diseases contributed 25% of mortality in the early 20th century in England, which decreased to 1% by the time antibiotics were widespread in the mid‐20th century (Smith et al. 2012). In both human and veterinary medicine, antibiotics stand out as one of the most powerful therapeutic choices for addressing infectious diseases caused by bacterial agents (Aminov 2017). Antibiotics are categorized by their distinct chemical structures and unique therapeutic activities related to their structures; thus, MoA is a key parameter for categorizing antibiotics (Etebu and Arikekpar 2016). As such, this paper will summarize the different classes of antibiotics based on the following MoA: (i) cell wall targeting, (ii) protein biosynthesis inhibition, and (iii) DNA replication inhibition (Kapoor et al. 2017). Table 3 summarizes antibiotic resistance mechanisms of some strains of LAB from different food sources reported in the past 5 years.

**TABLE 3:** Summary of antibiotic resistance mechanisms in lactic acid bacteria.

Antibiotic class/target	Mode of action	Resistance mechanism	Resistance genes	Example LAB strains
Protein synthesis inhibitors	Bind to 30S or 50S ribosomal subunits, disrupting initiation, elongation, or termination	Intrinsic resistance due to absence of cytochrome‐mediated transport and membrane impermeability; acquired resistance via HGT or mutation	*tet*(*M*), *tet*(*W/N/W*), *tet*(*K*), *erm*(*B*)	*Lpb*. *plantarum*, *Lcb*. *rhamnosus*, *Len*. *buchneri*, *Lb*. *helveticus*, *Lc*. *casei*, *Lcb*. *paracasei*
β‐lactams (Ampicillin)	Bind PBPs, preventing peptidoglycan cross‐linking	Altered PBPs; some strains show phenotypic resistance despite general susceptibility	Not specified	*Lim*. *fermentum*, *Lp*. *plantarum*, *Lg*. *salivarius*, *L*. *crispatus*
Glycopeptides (Vancomycin)	Bind D‐Ala‐D‐Ala in peptide chains, inhibiting cell wall synthesis	Intrinsic resistance via D‐Ala‐D‐Lac substitution; absence of typical resistance genes (*vanA*, *vanB*)	None detected (intrinsic resistance only)	Most *Lactobacillus* spp.
DNA replication inhibitors	Inhibit DNA gyrase and topoisomerase IV, disrupting DNA supercoiling and replication	QRDR mutations (e.g., *gyrA*, *parC*); efflux pump activity	*gyrA*, *parC*	*Lb*. *delbrueckii*, *Lp*. *plantarum*, *Lb*. *acidophilus*

*Note:* Resistance profiles may vary between strains even within the same species due to genetic plasticity and environmental origin.

Abbreviations: HGT, horizontal gene transfer; PBP, penicillin‐binding protein; QRDR, quinolone resistance‐determining region.

#### 3.1.1 Inhibitors of Protein Synthesis

Protein synthesis is crucial for bacterial and mammalian cell survival, which functions as a favorable target for the antibiotic mode of action. Antibiotics can disrupt the initiation, elongation, or termination steps of protein synthesis by binding to either the 30S or 50S ribosomal subunits in the bacterial 70S ribosome (Ullah and Ali 2017). Aminoglycosides interact with the 30S ribosomal subunit, disrupting peptide elongation and causing errors in mRNA translation, which impacts protein functionality, including those involved in cell membrane permeability. LAB exhibit intrinsic resistance to aminoglycoside antibiotics due to the absence of cytochrome‐mediated electron transport (Dušková et al. 2021; Gueimonde et al. 2013), which facilitates drug uptake and membrane impermeability (Duche et al. 2023). Aminoglycoside resistance genes are commonly identified in LAB isolated from farm environments, contributing to the spread of pathogenic bacteria within the gut microbiota via HGT and increased tolerance through structural modifications (Jaimee and Halami 2016). High MICs and phenotypic resistance have been reported in some LAB strains *Lacticaseibacillus rhamnosus* (*Lcb*. *rhamnosus*), *Lactiplantibacillus plantarum* (*Lpb*. *plantarum*), *Limosilimosilactobacillus fermentum* (*Lim*. *fermentum*), *Ligilactobacillus salivarius* (*Lig*. *salivarius*), and *Lentilactobacillus buchneri* (*Len*. *buchneri*) tested against streptomycin, gentamicin, and kanamycin (Rojo‐Bezares et al. 2006; Stefańska et al. 2021).

Tetracyclines bind to the A site of the 30S subunit, preventing tRNA from binding to ribosomes (Chopra and Roberts 2001). Tetracycline resistance in LAB is prevalent, with the most common resistance genes being tet(M) and tet(W/N/W) (Ma et al. 2021). The tetracycline resistance gene *tetK* has been identified in *Len*. *buchneri* (Anisimova and Yarullina 2019), while the *tetW* gene was found in three strains of *Lb*. *helveticus* (Guo et al. 2017). Additionally, the *tetM* gene, located within the Tn916 transposon, was identified in *Lacticaseibacillus paracasei* (*Lcb*. *paracasei*) (Devirgiliis et al. 2011), when it was originally found in *Enterococcus faecalis* , suggesting potential transfer through other mobile elements as indicated by Morandi et al. (2015). In a parallel manner, Yang and Yu (2019) demonstrated the successful transfer of the *tetM* and *tetS* genes from strains of *Lactobacillus delbrueckii subsp*. *bulgaricus* and *Lp*. *plantarum* to *L*. *monocytogenes* in a filter mating experiment.

Erythromycin resistance varies among *Lactobacilli*, with some strains showing resistance despite most *Lactobacilli* typically being susceptible, indicating that this resistance is acquired rather than intrinsic. For example, *Lacticaseibacillus casei* (*Lc*. *casei*) strains isolated from Mozzarella cheese were found to be resistant to erythromycin (de Souza et al. 2019). Additionally, the transfer of resistance genes such as *ermB* and multiple *tet* genes from *Lactobacilli* to pathogenic bacteria in the animal gut has been observed (Thumu and Halami 2019). Over time, the frequency of erythromycin resistance in *Lactobacilli* strains appears to have decreased, with recent findings indicating the occurrence of resistance primarily in specific regions (Vahabzadeh and Özpinar 2018).

LAB exhibited varying resistance toward chloramphenicol in a study by Yang and Yu (2019) where 71.8% of *S*. *thermophilus* strains obtained from fermented food samples from China were determined to be resistant to chloramphenicol, and all six of the isolated *Lp*. *plantarum* isolates were resistant. Similarly, *Lactobacillus*, *Enterococcus*, and *Leuconostoc* strains isolated from fermented Indian food were found to be highly resistant to chloramphenicol (Ojha et al. 2023). Meanwhile, *Lc*. *lactis* isolated from raw cow milk has been described to be susceptible to chloramphenicol (Hamdaoui et al. 2024). Similarly, only 5% of the 40 tested *E*. *faecalis* from milk cheeses in Italy showed resistance to chloramphenicol (Silvetti et al. 2019).

#### 3.1.2 Inhibitors of Cell Wall Synthesis

Cell walls are composed of peptidoglycans, which consist of repeating sugar polymers linked by short peptide chains (Zhydzetski et al. 2024). These chains include amino acids like glycine, which aid in cross‐linking between peptidoglycan strands. Penicillin‐binding proteins (PBPs) facilitate this cross‐linking, crucial for the stability and strength of the bacterial cell wall (Bertonha et al. 2023). The β‐lactam antibiotics, such as ampicillin, target PBPs due to their structural similarity to the PBP substrate D‐alanyl‐alanine, disrupting the peptidoglycan (Kim et al. 2023).

β‐lactam antibiotics typically affect *Lactobacillus* and *Pediococcus* species (Duche et al. 2023; Singla et al. 2018). Although *Lactobacilli* are generally susceptible to these antibiotics (Anisimova and Yarullina 2019; Saini and Tomar 2017), several studies have reported ampicillin resistance in strains such as *Lim*. *fermentum*, *Lpb*. *plantarum*, *Lg*. *salivarius*, and *Lactobacillus crispatus* ( *L*. *crispatus* ) (Anisimova and Yarullina 2019; Dec et al. 2017; Guo et al. 2017). Moreover, glycopeptides, such as vancomycin, inhibit cell wall synthesis by binding to the D‐alanine portion of peptide chains, preventing cross‐linking (Zeng et al. 2016). Vancomycin is ineffective against these *Lactobacilli* because it binds weakly to their cell walls, which is due to the presence of D‐Ala‐D‐Lac, instead of the vancomycin target D‐Ala‐D‐Ala. Previous studies have demonstrated that genes responsible for vancomycin resistance in other bacteria (i.e., *vanA* and *vanB*) do not appear in those LAB, suggesting they have a natural mechanism for producing D‐Ala‐D‐Lac, making them inherently resistant to vancomycin (Selim 2022; Wang, Dong, et al. 2022).

#### 3.1.3 DNA Replication Inhibitors

Quinolones disrupt bacterial DNA replication by inhibiting the activities of type II topoisomerases, specifically DNA gyrase and topoisomerase IV, which introduce supercoiling into DNA (Collins et al. 2024). This inhibition leads to the accumulation of single and double‐strand breaks in DNA, preventing proper DNA unwinding and duplication, which are essential for bacterial cell division and survival (Bush et al. 2020; Lungu et al. 2022). Fluoroquinolones, such as ciprofloxacin, are a prominent subgroup of quinolone antibiotics that contain a fluorine atom attached to the central ring system, enhancing their activity against bacterial pathogens (Heeb et al. 2011).

Quinolone resistance in LAB typically arises from mutations in the quinolone resistance‐determining regions (QRDR) of genes encoding topoisomerases, such as *gyrA* and *parC*, and through the activity of efflux pumps (Das et al. 2020). For example, resistance to ciprofloxacin identified in strains of *Lb*. *delbrueckii ssp*. *bulgaricus*, *Lpb*. *plantarum*, and *Lb*. *acidophilus* has been linked to substitution mutations in the *parC* subunit of topoisomerase IV, with the typical substitution being Ser 80 replaced by Leu. Strains lacking these mutations often rely on efflux pumps for resistance. However, strains with mutations in QRDRs of *parC* and/or *gyrA* typically exhibit higher levels of resistance, often combining multiple resistance mechanisms to survive antibiotic exposure (Jiang et al. 2016). Numerous studies documented the existence of *gyrA* and *parC* genes in resistant *Lactobacilli* (Anisimova and Yarullina 2019; Guo et al. 2017), but in none of the instances, the mutations matched the usual ones mentioned previously, indicating the existence of other amino acid substitutions in the DNA gyrase gene (Li et al. 2015). In addition, it remains unclear whether resistance was a consequence of these modifications. This uncertainty points to a significant research gap: despite observed resistance phenotypes and genetic markers, the precise molecular mechanisms regulating ciprofloxacin resistance in *Lactobacilli* have yet to be elucidated, and further investigation is required (Colautti et al. 2022) (Table 4).

**TABLE 4:** Antibiotic susceptibility profiles of some strains of lactic acid bacteria from food sources reported 2019–2024.

LAB	Antibiotic	Phenotypically resistant (R)/susceptible (S)	Resistance genes	Food source	References
*Streptococcus thermophilus*	Chloramphenicol, ciprofloxacin, erythromycin, clindamycin	S	N/A	Fermented milk products (China)	Li et al. (2019)
*Streptococcus thermophilus* ST2013N2	Tetracycline, ampicillin, streptomycin, gentamicin, ciprofloxacin, trimethoprim, kanamycin	R	strA, strB, sul1		
*Streptococcus thermophilus* ST2014N26	Streptomycin, ciprofloxacin, kanamycin	R	aph(3†)‐II, strA, strB		
*Lactobacillus* ssp.	Ampicillin, erythromycin, clindamycin	S	N/A		
*Lactobacillus delbrueckii ssp*. *bulgaricus* LB2013N19	Streptomycin, trimethoprim, kanamycin	R	sul1		
*Lactobacillus delbrueckii ssp*. *bulgaricus* LB2014N21	Streptomycin, gentamicin, tetracycline, chloramphenicol, ciprofloxacin, trimethoprim, kanamycin	R	aac(6′)‐aph(2″), aph(3″)‐III, tet(M)		
*Lacticaseibacillus casei* KN3	Erythromycin, imipenem, penicillin, amoxiclav, tigecycline	S	N/A	Kunu (Nigeria)	Duche et al. (2023)
	Clindamycin, cefoxitin, cefmetazole, ceftazidime, ciprofloxacin, trimethoprim, gentamycin, kanamycin, polymyxin‐b, tobramycin, methicillin, moxifloxacin, norfloxacin, nalidixic acid, gatifloxacin, teicoplanin, vancomycin	R	N/A		
*Lacticaseibacillus casei* GR4	Azithromycin, erythromycin, trimethoprim, imipenem, meropenem, tigecycline, tetracycline	S	N/A	Garri (Nigeria)	
	Cefoxitin, cefmetazole, ceftazidime, fusidane, gentamycin, kanamycin, polymyxin‐b, tobramycin, methicillin, moxifloxacin, norfloxacin, nalidixic acid, penicillin, teicoplanin, vancomycin	R	N/A		
*Lacticaseibacillus casei* AK1	Azithromycin, erythromycin, cotrimoxazole, trimethoprim, imipenem, meropenem, tigecycline	S	N/A	Fermenting Akpu (Nigeria)	
	Clindamycin, cefoxitin, cefmetazole, ceftazidime, ciprofloxacin, fusidane, gentamycin, kanamycin, tobramycin, methicillin, moxifloxacin, norfloxacin, nalidixic acid, gatifloxacin, penicillin, amoxiclav, teicoplanin, vancomycin, tetracycline	R	N/A		
*Enterococcus durans* NIFTEM 51	Ampicillin, chloramphenicol, ciprofloxacin, erythromycin, kanamycin, linezoid, rifampicin, tetracycline, trimethoprim	R	tet(M)	Home‐made Curd (India)	Ojha et al. (2023)
	Clindamycin, gentamycin, streptomycin, tigecycline, vancomycin	S	N/A		
*Lactobacillus delbreukii* NCDC 405	Ampicillin, chloramphenicol, ciprofloxacin, clindamycin, erythromycin, gentamycin, kanamycin, linezoid, streptomycin, trimethoprim, vancomycin	R	N/A	Cheese (India)	
	Rifampicin, tetracycline, tigecycline	S	N/A		
*Lactobacillus delbreukii* NCDC 184	Ampicillin, chloramphenicol, kanamycin, streptomycin, tetracycline, trimethoprim, vancomycin	R	N/A	N/R	
	Ciprofloxacin, clindamycin, erythromycin, gentamycin, rifampicin, linezolid, tigecycline	S	N/A		
*Lacticaseibacillus rhamnosus* NCDC 24	Ciprofloxacin, erythromycin, kanamycin, streptomycin, trimethoprim, vancomycin	R	N/A	N/R	
	Ampicillin, chloramphenicol, clindamycin, gentamycin, linezolid, rifampicin, tetracycline, tigecycline	S	N/A		
*Lactiplantibacillus plantarum* M27	Tetracycline, chloramphenicol, erythromycin	R	cat‐TC	Regional Cheese (Poland)	Nalepa and Markiewicz (2022)
*Lactiplantibacillus plantarum* M41	Tetracycline, erythromycin, chloramphenicol	S	erm(B), cat‐TC		
*Lactiplantibacillus plantarum* BFM 29a	Tetracycline, chloramphenicol	R	tet(M), erm(B), cat‐TC		
	Erythromycin	S	N/A		
*Lactobacillus delbrueckii* M22	Chloramphenicol	R	N/A		
	Tetracycline, erythromycin	S	N/A		
*Lactobacillus delbrueckii* Lb49	Tetracycline	R	cat‐TC		
	Chloramphenicol, erythromycin	S	N/A		
*Lactobacillus delbrueckii* P107	Tetracycline, chloramphenicol, erythromycin	S	tet(M)		
*Lacticaseibacillus casei* BFM 24d	Tetracycline, chloramphenicol, erythromycin	S	N/A		
*Lacticaseibacillus casei* BFM 26d	Erythromycin	R	tet(M), erm(B), cat‐TC		
	Tetracycline, chloramphenicol	S			
*Lacticaseibacillus paracasei* B5	Tetracycline, chloramphenicol, erythromycin	S	erm(B)		
*Lacticaseibacillus paracasei* P96	Tetracycline, chloramphenicol, erythromycin	R	tet(L), cat‐TC		
*Enterococcus faecium* P32	Tetracycline	R	cat‐TC		
	Chloramphenicol, erythromycin	S	N/A		
*Leuconostoc mesenteroides* ZT6	Nalidixic acid, clindamycin, tetracycline, novobiocin, neomycin, erythromycin, streptomycin, oxacillin	R	N/A	Raw milk and kefir grains (Turkey)	Akpinar and Yerlikaya (2021)
*Lacticaseibacillus paracasei* SMM1	Bacitracin, novobiocin	R	N/A		
*Lactiplantibacillus plantarum* R3	Gentamycin	R	aac(6′)‐aph(2″)	Yogurt (China)	Yang and Yu (2019)
*Lactiplantibacillus plantarum* R18	Gentamycin, sulfamethoxazole	R	aac(6′)‐aph(2″), sulI		
*Lactiplantibacillus plantarum* R41	Tetracycline	R	tetS	Fermented dairy drink (China)	

*Note:* Resistance or susceptibility classifications for the listed LAB strains are based on MIC values reported in the cited studies. MIC reflects the concentration needed to inhibit visible bacterial growth and is used to assess resistance. However, it does not account for antibiotic tolerance, which may survive treatment without genetic resistance.

Abbreviations: N/A, not available; N/R, not reported.

The differences in AR among various LAB strains and species can be largely attributed to the presence of mobile genetic elements carrying resistance genes. In Nigerian fermented foods, for instance, *Lacticaseibacillus casei* (*Lcb*. *casei*), *Levilevilactobacillus brevis* (*Lev*. *brevis*), and *Lcb*. *paracasei* strains were found to be susceptible to tetracycline, despite carrying the *tetM* resistance gene and one strain harboring the *Tn916* transposon, suggesting that the presence of resistance genes does not always translate to phenotypic resistance (Duche et al. 2023). In contrast, *Weisella* strains from various environmental sources, which carried tetracycline resistance genes (tetK, tetS, tetM, and tetO), were present in all strains that exhibited 100% resistance to tetracycline (Fhoula et al., 2022). These findings highlight a frequent mismatch between genotypic and phenotypic resistance in LAB, highlighting the need for both genetic and phenotypic assessments when evaluating the safety of LAB.

Tetracycline resistance has also been observed in LAB strains from raw milk cheese in Italy, but the detected resistance was not transferred during yogurt production and storage (Morandi et al. 2015). Similar resistance patterns were found in food products, where *Lb*. *acidophilus*, *Lcb*. *casei*, and *Limosilactobacillus reuteri* (*Lim*. *reuteri*) from Chinese fermented milk showed phenotypic and genotypic resistance to tetracycline and did not spread their resistance to other bacteria through filter mating assay studies (Guo et al. 2017). Interestingly, *Streptococcus thermophilus* strains, previously considered as sensitive, developed resistance to tetracycline in Chinese fermented milk and commercial yogurt, with 31 out of 39 strains showing resistance to multiple antibiotics including tetracycline (Yang and Yu 2019).

Resistance to erythromycin was prominent in a study by Ojha et al. (2023), where 86% of LAB strains isolated from Indian fermented foods exhibited resistance, potentially due to HGT. Similarly, resistance genes for chloramphenicol are often located on mobile genetic elements in LAB, which may explain the varied resistance patterns observed in different environments (Nalepa and Markiewicz 2022). Quinolone resistance is also notable, with 76.5% of LAB strains from Indian fermented foods showing resistance to ciprofloxacin, likely driven by intrinsic resistance mechanisms (Ojha et al. 2023).

### 3.2 Intrinsic Versus Acquired Resistance Mechanisms in LAB

LAB can exhibit resistance to an antimicrobial through intrinsic (natural) or acquired (transmissible) methods (Zheng et al. 2024). Intrinsic resistance refers to the inherent ability of a bacterial species or genus to endure in the presence of an antimicrobial agent as a result of its intrinsic resistance traits common to all strains of the species (Alonso et al. 2024). This type of resistance is generally consistent, inheritable, and predictable, with minimal potential for horizontal spread between different bacterial species. For probiotic strains, intrinsic AR is viewed as an advantageous trait, especially when administered in addition to antibiotics to guarantee the survival of probiotic bacteria in the gut and supports gut microbial diversity (Duche et al. 2023).

**FIGURE 2:**
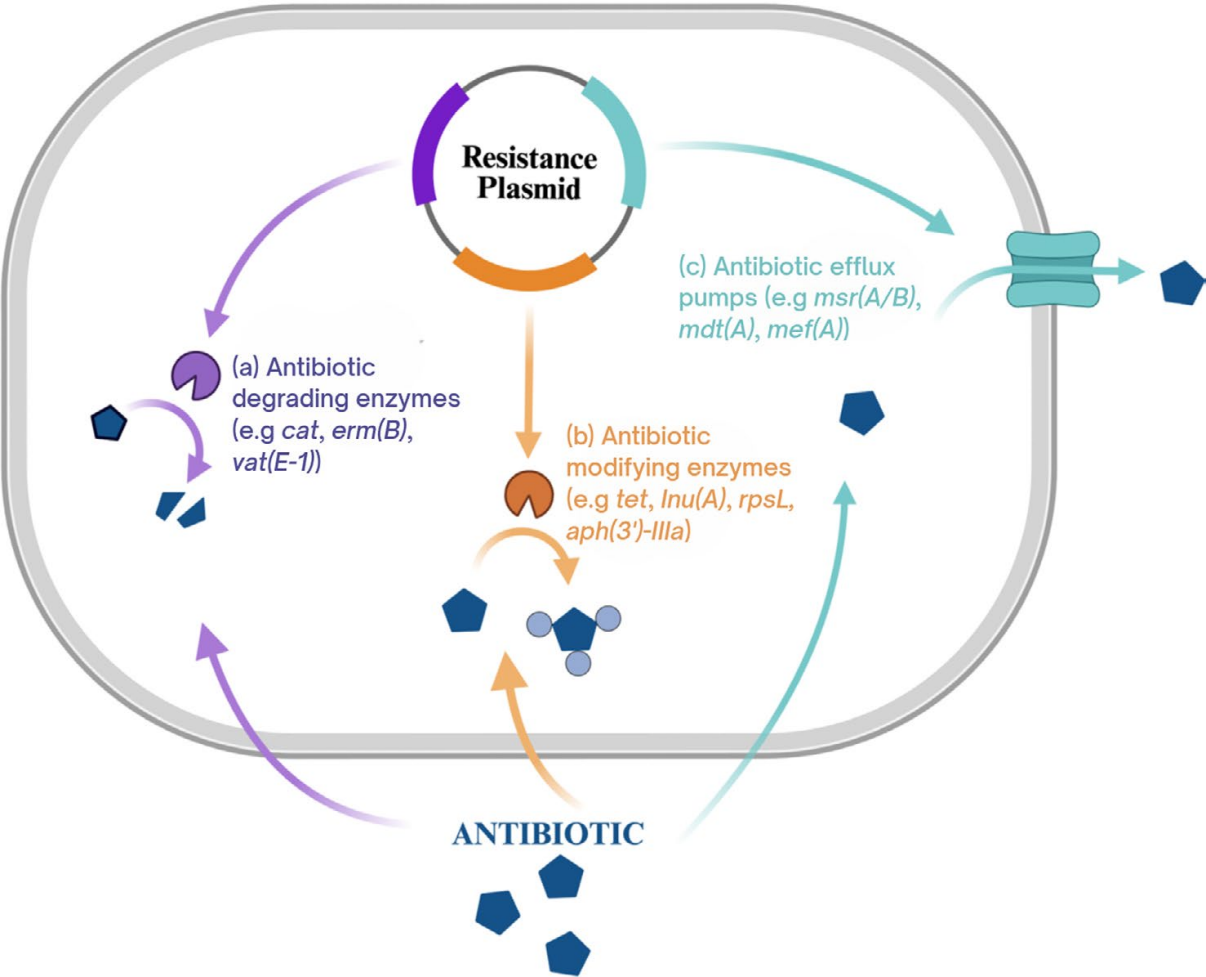
Mechanisms of intrinsic AR in LAB: (a) Enzymatic degeneration of intra‐ or extra‐cellular antibiotics; (b) alteration of the antibiotic by enzymatic complexes that inhibit the antibiotic‐target interaction; and (c) a decrease in intracellular antibiotic concentration through the activation of efflux pumps or modifications in cell wall permeability (Sharma et al. 2014).

As shown in Figure 2, LAB can develop intrinsic resistance to antibiotics through several mechanisms. One primary method is by harboring plasmids that produce enzymes capable of either neutralizing or breaking down antibiotics, making them ineffective. For example, certain LAB strains produce β‐lactamases that hydrolyze the β‐lactam ring of antibiotics like penicillin, leading to resistance (Girlich et al. 2007). Another resistance mechanism involves the use of active efflux pumps, controlled by plasmids carrying resistance genes, that expel antibiotics from bacterial cells, reducing the internal concentration of the antibiotics. An example is the LmrA transporter in *Lactobacillus delbrueckii ssp*. *lactis*, which has been linked to resistance against a broad range of antibiotics (Wacher‐Rodarte Mdel et al. 2016). Thus, reduced drug permeability or enhanced expulsion of drugs from the cell surface plays a role in AR. In addition, some bacteria alter their target sites, such as the binding proteins on their cell walls, which are crucial for antibiotic action. These modifications prevent antibiotics from binding effectively, leading to resistance against various drug classes, including aminoglycosides, fluoroquinolones, tetracyclines, sulfonamides, macrolides, beta‐lactams, and vancomycin (Sharma et al. 2014).

Aquired resistance occurs when a strain of a typically susceptible species gains resistance to an antimicrobial drug (European Food Safety Authority (EFSA) 2005). This type of resistance is found in certain strains within a species and results either from mutations in the bacterial genome or from the acquisition of additional genes encoding resistance mechanisms. It is particularly prevalent in microorganisms that inhabit environments frequently exposed to antibiotics, such as human and animal intestines. This type of resistance is found in certain strains within a species and occurs through various methods, including HGT, conjugation, and plasmid acquisition (Sagar et al. 2019). Consequently, acquired resistance poses a greater risk for HGT, as the resistance genes are often located on mobile genetic elements like plasmids and transposons (Devirgiliis et al. 2011).

## 4 Evolution and Spread of AR Among LAB

AR develops when bacteria gain the ability to withstand the effects of antibiotics, a process closely tied to the excessive or uncontrolled use of antibiotics in agriculture and animal breeding (Acar and Röstel 2001; Thompson et al. 2023). Antibiotic use in animal husbandry began in the 1940s and became widespread by the 1950s when it was discovered that subtherapeutic doses could enhance growth and feed efficiency in livestock (Graham et al. 2007). This practice, known as antibiotic growth promotion (AGP), was commonly applied in poultry, swine, and cattle farming (Peng et al. 2014; Sapkota et al. 2007). Notably, Albernaz‐Gonçalves et al. (2022) highlight that poor welfare in intense livestock environments is both an ethical issue and a driver of antibiotic overuse: stressed, immunocompromised animals housed in crowded, barren environments routinely receive antibiotics to compensate for substandard conditions. By the 1960s and 1970s, concerns emerged over the potential for antibiotic use in agriculture to contribute to the development of antibiotic‐resistant bacteria that could impact human health (McKenna 2017). In response, several governments and regulatory bodies initiated policy changes. The European Union progressively restricted antibiotic use in feed and implemented a complete ban on antibiotics for growth promotion by 2006 (Casewell et al. 2003). In the United States, regulatory action was slower, but the FDA issued Guidance for Industry #213 in 2017, which required veterinary oversight for medically important antibiotics and banned their use for growth promotion (FDA 2013). These changes have spurred interest in alternatives to antibiotics, such as probiotics, prebiotics, and vaccination strategies, to support animal health while minimizing antimicrobial resistance (Council et al. 1999). In 2017, 46% of China's total antibiotics (162,000 tons) were used in animal production, while in 2012, the U.S. FDA reported that 82% of 17,900 tons of antibiotics were similarly used in animal production (Ying et al. 2017).

This overuse leads to antibiotic residues (active substances, excipients, and degradation products) in food and the environment. Such residues expose microbes to subtherapeutic doses, promoting the emergence of resistant bacteria (Arsène et al. 2021). These resistant bacteria and their resistance genes can spread through shared ecosystems involving humans and animals, transferring via direct contact, food, water, or environmental exposure. Regarding environmental exposure, research involving *Lpb*. *plantarum* and *Lcb*. *paracasei* strains isolated from the fuel ethanol industry revealed that continuous exposure to penicillin G led to the development of resistance (Walter et al. 2019). Thus, persistent antibiotic exposure in shared ecosystems can drive the selection and spread of resistant bacteria and resistance genes, highlighting the importance of monitoring sources of antimicrobial resistance.

**FIGURE 3:**
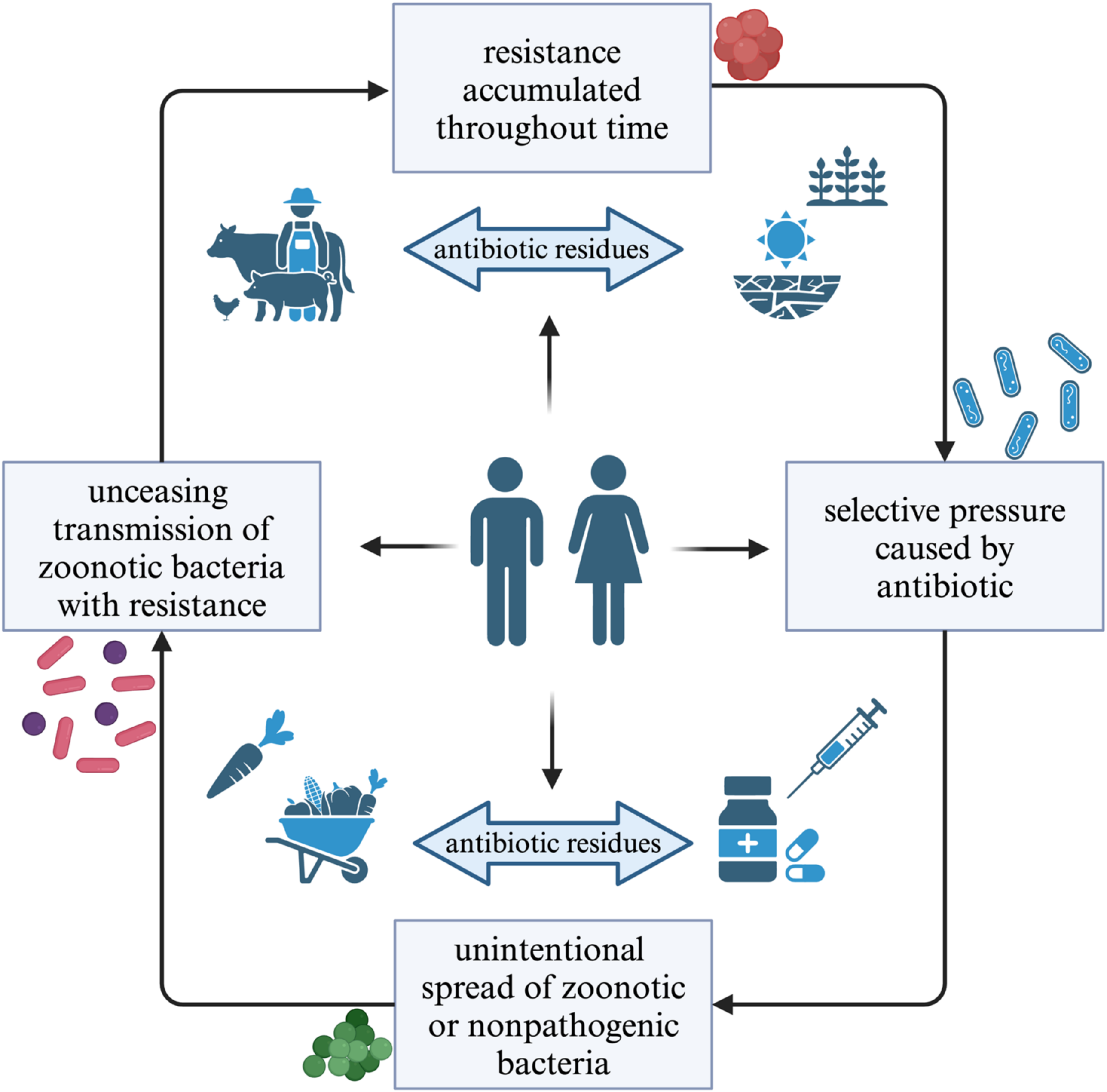
Overview of the mechanisms underlying the spread and development of AR. In shared ecosystems between humans and animals, resistant bacteria and their resistance genes can proliferate through direct contact, food, drink, or ambient exposure. Furthermore, microbial ecosystems can be upset by lingering antibiotic residues in soil and water, which promote the spread of resistance (Spînu et al. 2018).

Additionally, antibiotic use in livestock farming, particularly in chicken feed, has been identified as a major driver of AR development (Koch et al. 2017). In a study conducted by Song et al. (2022), chicken farms where antibiotics are added to the feed had higher levels of AR genes and mobile genetic elements compared to the farms without antibiotic use. In addition, airborne AR genes found in these farms were more concentrated, indicating that the indoor air of chicken farms can act as a reservoir for resistant genes (Song et al. 2022). A study of LAB isolated from broiler chicken feces in Malaysia found that bacteria exhibited resistance to multiple antibiotics, which was attributed to the long‐term use of antibiotics as growth promoters in poultry farming (Shazali et al. 2014). In another study, researchers found that *Lactobacillus* strains isolated from poultry probiotic products carried AR genes and confirmed phenotypic resistance against tested antibiotics. They also observed through co‐culture experiments the transfer of those genes to *E*. *coli*, which exhibited similar resistance patterns to its gene donor (Rokon‐Uz‐Zaman et al. 2023). This finding highlights a serious risk to consumers, as resistant bacteria or resistance genes originating from animal probiotics can potentially enter the human food chain, contributing to the spread of antibiotic resistance in human gut microbiota. As a result, the threat of AR increases and poses a risk to all species due to the constant interactions between humans, animals, food, and the environment (Figure 3).

AR originates and spreads as a result of the immense genetic diversity among bacterial cells, which allows frequent mutations and gene transfers. Novel resistance genes often occur with fitness costs, such as ineffective expression or disruption of essential cellular functions, and therefore, are maintained only under strong selection pressures (Larsson and Flach 2022). These genes can emerge through mechanisms like efflux pump upregulation, cell wall modifications, enzyme expression for degradation, protection of antibiotic targets, or alternative enzymatic functions (Zhang and Cheng 2022).

HGT plays a vital role in the spread of antibiotic resistance genes, allowing them to move between bacterial species under stress conditions (Liu et al. 2021). This transfer is more frequent among closely related bacteria within the same microenvironment. Nicolas et al. (2007) analyzed 401 core genes, wherein 40% of which may have been transferred between *Lb*. *acidophilus* and *Lb*. *johnsonii* (both isolated from the GI tract), indicating that HGT most likely occurred. In addition, high‐risk environments that may lead to HGT include those with elevated antibiotic levels, such as human and animal microbiomes during treatment, intensive aquaculture, and polluted areas. The presence of sub‐lethal antibiotic concentrations can promote gene transfer and resistance development as well (Larsson and Flach 2022).

The evolution of AR involves bacterial adaptation to stressful environments and compensatory mutations that reduce fitness costs (Durão et al. 2018). In a study conducted by Lin et al. (2018), rifampin‐resistant *E*. *coli* strains exhibited reduced fitness costs under low‐nutrient conditions, allowing them to maintain a competitive advantage against wild‐type cells. This finding highlights the importance of studying the bacteria's adaptation processes, as they are important for understanding health risks and creating strategies to control the spread of resistance genes from the environment to pathogens. In addition, identifying high‐risk scenarios and environments is crucial for preventing the spread of resistant bacteria and the incorporation of resistance genes into human pathogens (Pal et al. 2016). Such high‐risk environments may include intensive livestock farming where antibiotics are routinely used, hospitals and long‐term care facilities with high antibiotic usage, wastewater treatment plants where antibiotic residues and resistant microbes accumulate, and food processing environments where cross‐contamination between probiotic and pathogenic bacteria may occur.

AR genes spread among bacteria through several key mechanisms: transformation, transduction, and conjugation. Transformation involves bacteria taking up foreign genetic material from their environment. Transduction occurs when bacteriophages incorporate bacterial DNA during replication and transfer it to other bacterial cells upon infection. Conjugation, the most common method, requires direct cell‐to‐cell contact where DNA, often carried by plasmids, is transferred between bacteria. Notably, mobile genetic elements such as plasmids, transposons, and bacteriophages play crucial roles in these processes (van Reenen and Dicks 2011).

LAB can also facilitate the transfer of AR genes to other pathogenic bacteria, thus posing significant public health risks (Devirgiliis et al. 2011; van Reenen and Dicks 2011). For instance, *Streptococcus* species such as *S*. *thermophilus* , *S*. *agalactiae* , and *S*. *pneumoniae* can be problematic due to their genomic instability. This instability, often attributed to the absence of key genes like *recQ* helicase, *sbcC*, and *sbcD*, makes these bacteria more prone to acquiring AR genes through HGT (Noda et al. 2019). Additionally, phage‐mediated transfer contributes to the spread of AR genes. In particular, phages can facilitate horizontal gene transfer among microbes by integrating their DNA into the bacterial chromosome (Balcazar 2014). Huang et al. (2023) have recently identified prophages in streptococci species such as *S*. *agalactiae* and *S*. *suis* which carry various AR genes. In addition, *E*. *faecalis* found in the human gut and dry sausage carries conjugative multi‐resistance plasmids like pRE25, which can transfer resistance to other bacteria (Schwarz et al. 2001). Consequently, the high cell density and diversity in the gut ecosystem enhance the transfer of these multiresistance plasmids, promoting the spread of resistance among Gram‐positive bacteria (Arias et al. 2010). Although these findings demonstrate the potential for ARG, a significant research gap remains. Specifically, the frequency at which LAB transfer ARGs to pathogenic bacteria in vivo is still unknown. Addressing this gap is essential for accurately evaluating the public health risks associated with the spread of resistance and probiotics.

## 5 Navigating the Challenges of Antibiotic Resistance

### 5.1 The Decline of New Antibiotics in the Midst of Rising Resistance

The antibiotic industry is in a state of significant decline, struggling to keep pace with the growing threat of AR. According to the World Health Organization (2022), the development of new antibiotics and antimicrobial treatments has not matched the urgent need, with only a few new antibiotics being approved in recent years, most of which belong to existing classes and are therefore vulnerable to resistance. This innovation gap is compounded by economic challenges, as the profitability of developing new antibiotics is low compared to other therapeutic areas. High development costs, low profitability, and the necessity to conserve new antibiotics as last‐resort options have led many large pharmaceutical companies to exit the market, leaving small and medium‐sized enterprises (SMEs) to struggle financially, often to the point of bankruptcy (Årdal et al. 2020). According to Ciabuschi and Lindahl (2018), the number of large firms involved in antibacterial drug discovery has dropped from 25 in 1980 to 4 in 2018, following the closure of additional R&D programs. Despite the increasing prevalence of antibiotic‐resistant bacteria, which are liable for hundreds of thousands of deaths annually, regulatory and scientific hurdles coupled with the impact of the COVID‐19 pandemic have made it difficult to bring new antibiotics to market (Brüssow 2024). The result is a decline in the effectiveness of current antibiotics and an increasing vulnerability of the global population to resistant infections (Okeke et al. 2024). Without significant investment in research and development, particularly in innovative approaches, the situation is likely to worsen, threatening the foundation of modern medicine.

### 5.2 Advances in Bioinformatics and Their Use in Predicting and Identifying ARGs

Recent advances in bioinformatics, metagenomics, and artificial intelligence (AI) have significantly enhanced the identification and prediction of ARGs (Behling et al. 2023). Metagenomic profiling, which involves sequencing bacterial communities without the need for culturing, enables the detection of ARGs across diverse microbial populations. By clustering genes based on sequence similarity, researchers can map them against ARG databases like CARD and ResFinder to identify resistance genes (Boolchandani et al. 2019). The ResFinder and PointFinder databases offer precise antimicrobial resistance phenotypes for specific species and antibiotic combinations by detecting ARGs and resistance‐related mutations. These tools are highly accurate for well‐studied organisms and antibiotics but are limited when applied to less‐researched antibiotics due to insufficient knowledge of the associated resistance mechanisms (Aytan‐Aktug et al. 2020). To address this, advanced AI‐based tools such as Fragmented Antibiotic Resistance Gene Identifier (fARGene) and DeepARG have been developed for predicting novel ARGs with limited similarity to known genes, using hidden Markov models and deep learning. These tools can detect new resistance genes directly from metagenomic data without assembly. For example, in a study conducted by Berglund et al. (2019), more than 80% of the novel AR genes predicted by fARGene were experimentally validated to induce a resistance phenotype in *E*. *coli*. Machine learning (ML) has been applied to identify complex patterns in metagenomic data, aiding in the identification of ARGs and resistance profiles (Rahman et al. 2018). The horizontal transfer of ARGs between bacterial genomes can also be explored through metagenomic approaches, providing valuable insight into how resistance spreads. Ku et al. (2025) investigated the role of bacteriophages in the spread of β‐lactam resistance gene in river water samples. Using metagenomic techniques to analyze phage sequence from the NCBI database and environmental *E*. *coli* isolates, the researchers identified the presence of the blaCTX‐M gene within bacteriophage populations in the water. Subsequent cloning of phage DNA into ampicillin‐sensitive *E*. *coli* resulted in the acquisition of resistance, indicating the occurrence of HGT. AI and ML tools are increasingly being used for drug discovery as well. For instance, ML has been used to repurpose existing drugs, such as the discovery of Abaucin, an antibacterial compound identified through AI that is effective against multidrug‐resistant *Acinetobacter baumannii* PEVuZE5vdGU(de Abreu et al. 2020). This suggests that AI and bioinformatics will continue to play a key role in predicting and combating AR in the future.

Despite their powerful capabilities, the aforementioned strategies may face several limitations. One key challenge is the reliance on existing databases, which often leads to bias toward well‐characterized organisms and resistance mechanisms. For instance, Boolchandani et al. (2019) note that ARG identification is hindered when metagenomic sequences originate from poorly studied environments or organisms lacking comprehensive representation in databases such as CARD and ResFinder. Similarly, Zankari et al. (2017) emphasize that while tools like PointFinder provide accurate results for well‐known ARGs, their effectiveness diminishes when applied to rare or novel resistance genes, particularly those conferring resistance to less commonly used antibiotics. In the context of AI tools, DeepARG and fARGene, while effective at predicting novel ARGs, can also produce false positives due to model overfitting or the presence of conserved non‐resistance‐related domains (Arango‐Argoty et al. 2018). Lastly, although machine learning tools offer great promise, they require extensive, high‐quality labeled datasets for training, which are not always available, limiting model generalizability (Rahman et al. 2018). These constraints highlight the need for continued development of databases, experimental validation, and more sophisticated algorithms to fully realize the potential of these technologies in AR surveillance.

### 5.3 Novel Strategies to Mitigate AR in LAB

Combination therapy, which involves administering an antibiotic together with a mechanism inhibitor, is highly effective in overcoming resistance mechanisms (Drawz et al. 2014). Inhibiting efflux pumps has proven to be successful in suppressing resistant phenotypes. For instance, using berberine, an efflux inhibitor from Corydalis Tuber, with ciprofloxacin, a quinolone antibiotic, has been shown to suppress resistance in *S*. *aureus* (Seo et al. 2024), while FDA‐approved drugs like raloxifene and pyrvinium act as strong NorA (efflux pump) inhibitors, boosting ciprofloxacin activity and enhancing the sensitivity of *S*. *aureus* against multiple antibiotics (Mahey et al. 2021). Efflux pump inhibitors work by allowing intracellular drug accumulation, complementing antibiotic action, and avoiding multidrug resistance (Kourtesi et al. 2013; Lamut et al. 2019). Furthermore, inhibitors target resistance mechanisms without harboring antimicrobial potential themselves, thus avoiding the acquisition of new resistance (Pu et al. 2024).

Bacteriocins, which are antimicrobial peptides generally produced by lactic acid bacteria, have been utilized in the food industry for over 50 years and are FDA‐approved as GRAS. One well‐known example is nisin, a bacteriocin produced by *Lactococcus lactis* subsp. *lactis*, which has been approved by the FDA for use as a food preservative due to its safety and effectiveness against Gram‐positive bacteria (Putri et al. 2024). Bacteriocins and bacteriocin‐like inhibitory substances (BLISs) exhibit broad or narrow spectrum effects against antibiotic‐resistant bacteria, presenting a promising alternative to antibiotics (Verma et al. 2022). Shokri et al. (2014) found a BLIS produced by *E*. *faecium* DSH20 in large amounts that was effective specifically against vancomycin‐resistant *Enterococci*. Similarly, El‐Gendy et al. (2021) discovered BLIS enterocin OS13 from food‐isolate of *E*. *faecalis* OS13 that displayed antagonistic effects on AR *E*. *faecalis* and *E*. *faecium* *s*trains.

Various bacteriocins produced by enterococci have been reported in the literature to inhibit other multidrug (MDR) and AR enterococci. One such report is from Phumisantiphong et al. (2017) who found a bacteriocin from *E*. *faecalis* EF 478 that exhibited inhibitory effects against MDR‐ and vancomycin‐resistant (VR) enterococci. Additionally, Kwandee et al. (2023) confirmed the effectiveness of a possible bacteriocin produced by Rhizobium sp. AG207R isolated from ginger roots against VR *E*. *faecalis* . Saelim et al. (2015) reported the inhibition effect of ce5‐1 bacteriocin from *E*. *faecium* ce5‐1 against VR strains of *E*. *faecalis* , *E*. *faecium* , and *E*. *gallinarum* . The lethal effect achieved against *E*. *faecalis* was primarily due to damage inflicted by the formation of spores and filaments on the cell wall. Furthermore, Gallocin D, a bacteriocin isolated from *Streptococcus gallolyticus* LL009, has been found to be effective against VR (Hill et al. 2020).

Prebiotics, such as dietary fibers, are also essential for reducing antimicrobial resistance. For instance, a 2022 study showed that acacia fiber supplementation significantly reduced colonization of extended‐spectrum beta‐lactamase (ESBL)‐producing *E*. *coli* in antibiotic‐treated mice by nourishing native bacteria to produce colicin M, which inhibited pathogenic strains (Maeusli et al. 2022). Similarly, residue fiber intake reduces antibiotic‐induced dysbiosis, helping maintain gut redox balance, thus supporting microbiome recovery during antibiotic therapy (Safarchi et al. 2025). Beyond resilience, higher long‐term fiber intake in healthy adults correlates with lower levels of antibiotic resistance genes, highlighting their suppression via dietary means (Oliver et al. 2022). Prebiotic fibers fuel SCFA‐producing commensals, which improve gut barrier function, lower colonic pH, and outcompete pathogens, thereby reducing the ability for the survival of resistant strains (Safarchi et al. 2025).

Targeting ARGs through CRISPR‐Cas systems offers a promising approach within LAB. In a study conducted by Rodrigues et al. (2019), a CRISPR‐based antimicrobial system using pheromone‐responsive plasmids was developed to target erythromycin resistance genes in *E*. *faecalis* . A reduction in erythromycin resistance was achieved in enterococci sourced from mouse intestines (i.e., modeled after human intestinal conditions). Despite the low overall delivery frequency of the CRISPR‐Cas system due to low conjugation rates, recipient cells displayed a complete loss of erythromycin resistance. This strategy has also been applied successfully in *B*. *animalis* subsp. *lactis* to eliminate the tetracycline resistance gene *tetW* (Arigoni 2008). Overall, the utilization of CRISPR‐Cas systems for mitigating AR is an efficient and cutting‐edge technique to reduce and prevent the transfer of AR genes (Hidalgo‐Cantabrana et al. 2017).

As previously discussed, conjugation‐based delivery methods often exhibit low efficiency in vivo within gut environments, which drop further when taxonomic distance increases between donor and recipient bacteria (Neil et al. 2021; Wongpayak et al. 2021). Transfer rates also greatly decrease across taxonomic boundaries in mouse models and liquid mating assays. In addition, plasmid stability is compromised by segregational loss and fitness costs, where, without selection pressure, cells may lose the CRISPR‐Cas plasmid over time, especially non‐conjugative ones (Dorado‐Morales et al. 2021; Prensky et al. 2021). Although conjugative plasmids persist better than non‐conjugative ones, their stability depends on a balance between transfer frequency and host fitness costs. To counteract this loss, maintenance systems like toxin–antitoxin (TA) systems can aid plasmid retention but do not fully eliminate the burden (Bethke et al. 2024; Dey et al. 2023).

These challenges necessitate optimized delivery vectors, improved maintenance systems, and microbiome‐aware engineering strategies for CRISPR‐based antimicrobial therapies.

Another promising genome editing tool for targeting ARGs has been developed by Zuo et al. (2019) using an inducible self‐destruction plasmid (IPSD). This replicative plasmid separates the ARG and the replicon with the help of a site‐specific recombinase driven by an inducible promoter. This tool was successfully used for gene knockouts and knock‐ins in *Lactobacilli* and bifidobacteria, offering a simple and universal approach for genetic engineering across various bacterial species since it does not depend on transformation efficiency. When tested in *Bifidobacterium longum* IF3‐53 to knockout the tetracycline resistance gene *tetW*, the IPSD method achieved a 19% success rate for correct single‐crossover integration, resulting in mutants with significantly reduced tetracycline resistance. Moreover, the results demonstrate the potential of the IPSD plasmid as an effective genome engineering tool in bifidobacteria.

Another approach to alleviate AR in LAB is the use of antimicrobial nanomaterials, which offer high antimicrobial efficacy and diverse antibacterial mechanisms. For example, gold nanoparticles (Au NPs) especially have been referred to carry potential in nano‐scale antibiotics due to their cytocompatibility, high surface‐to‐volume ratio, and controlled surfaces (Nguyenova et al. 2024; Salesa et al. 2023; Zhang et al. 2019). As such, they have been modified by Wang, Zheng, et al. (2022) with aminophenol (AP) to mimic the antibacterial action of aminoglycosides by binding to the 16S ribosomal RNA (rRNA). The AP binding ability is strengthened by its amino group, which binds to the gold nanoparticle, while its hydroxyl group binds to the organism. Furthermore, the aminophenol‐coated nanoparticles attach to the rRNA by forming hydrogen bonds between their hydroxyl groups (i.e., those of the phosphoric acids and ribose on the rRNA and the aminophenol). The main antibacterial mechanisms observed were due to the inhibition of protein synthesis as a result of binding to the 16 s subunit of the ribosome and bacterial cell wall destruction, the latter of which affects MDR bacteria. In a parallel manner, Wang et al. (2023) developed a non‐toxic mercaptophenylboronic acid‐modified gold nanocluster, Au44(MBA)18, with the aim of eliminating VE *E*. *faecalis* . The nanocluster was shown to act against Gram‐positive bacteria by initially attaching to teichoic acid on the cell wall membrane, followed by binding to bacterial DNA, leading to bacterial cell wall destruction and the death of VE *E*. *faecalis* .

## 6 Conclusions and Future Perspectives

Today, the growing use of probiotics has sparked a significant interest in LAB and their associated health benefits. However, the concern of AR in LAB cannot be overlooked. While intrinsic resistance in LAB can potentially boost their survival under harsh conditions of antimicrobial environments, acquired resistance presents a more complex scenario. Although acquired resistance in LAB allows bacteria to survive under such conditions, it also risks transferring resistance genes to pathogens in the gut, leading to the spread of antimicrobial resistance and related health concerns. The risk is amplified even more critically as the probiotic industry continues to expand, with LAB being incorporated into various food products and dietary supplements. As a result, the potential for transferring AR genes to pathogenic bacteria goes up, undermining public health efforts to control infectious diseases.

Addressing this challenge requires regulatory frameworks to evolve alongside the expanding probiotic industry. Regulatory bodies such as the EFSA and the FDA have emphasized the need for robust safety assessments. Specifically, EFSA guidelines call for antibiotic susceptibility testing and further analysis of strains carrying resistance genes, particularly those on mobile genetic elements. Similarly, the FDA's GRAS framework emphasizes the importance of rigorous, evidence‐based safety evaluations to prevent the spread of AR through probiotic consumption.

Manufacturers must implement strict screening processes to identify and exclude AR strains. Recent advances in bioinformatics, metagenomics, and AI have revolutionized the identification and prediction of ARGs. By utilizing these tools, manufacturers can better understand the implications of using novel probiotics and optimize their formulations to reduce resistance risks, ensuring safer products for consumers. Meanwhile, consumers should advocate for clearer product labeling and transparency regarding microbial content to make informed health decisions.

In conclusion, a coordinated, multifaceted approach is essential. This includes rigorous safety measures by producers, informed consumer choices, strengthened regulatory oversight, and innovative technological applications. Just as important is active collaboration among researchers, industry stakeholders, regulators, and healthcare professionals to ensure transparent data sharing, harmonized safety standards, and ongoing risk monitoring. To uphold ethical standards and public trust, probiotic research must also follow transparent and standardized practices. Funding sources and affiliations should be fully disclosed, while independent laboratories must verify strain identity and safety before commercialization. All clinical and safety evaluations should be pre‐registered and report complete results to minimize publication and outcome‐reporting bias. Together, these coordinated scientific, ethical, and regulatory efforts will help preserve the benefits of probiotics while minimizing potential risks, ensuring they remain a safe and effective tool in advancing human health for future generations.

## Author Contributions

**Salma Sherif Refaat:** conceptualization (equal), formal analysis (equal), investigation (equal), methodology (equal), writing – original draft (equal). **Fatih Ortakcı:** investigation (equal), methodology (equal), writing – original draft (equal), writing – review and editing (equal). **Zeynep Akinan Erdem:** data curation (equal), formal analysis (equal), investigation (equal), methodology (equal), writing – original draft (equal). **Muhammed Zahid Kasapoğlu:** data curation (equal), methodology (equal), writing – original draft (equal). **Enes Dertli:** conceptualization (equal), data curation (equal), project administration (equal), supervision (equal), writing – original draft (equal), writing – review and editing (equal).

## Conflicts of Interest

The authors declare no conflicts of interest.

## Data Availability

Data will be made available upon request.
